# Analysis of World-Scale Mitochondrial DNA Reveals the Origin and Migration Route of East Asia Goats

**DOI:** 10.3389/fgene.2022.796979

**Published:** 2022-04-26

**Authors:** Weifeng Peng, Yiyuan Zhang, Lei Gao, Cailing Feng, Yujiao Yang, Bingyi Li, Lili Wu, Ali Wu, Shuping Wang, Xue Ren, Zehui Chen, Min Zhang, Danni Cai, Xin Wang, Mengqi Lv, Yitong Zhang, Simeng Li, Yunxia Zhang, Li Huang, Shiwei Li

**Affiliations:** ^1^ College of Life Science and Agronomy, Zhoukou Normal University, Zhoukou, China; ^2^ State Key Laboratory for Sheep Genetic Improvement and Healthy Production, Shihezi, China; ^3^ Zhoukou Hospital of Traditional Chinese Medicine, Zhoukou, China; ^4^ State Key Laboratory of Environmental Criteria and Risk Assessment, Chinese Research Academy of Environmental Sciences, Beijing, China; ^5^ Annoroad Gene Technology (Beijing) Co., Ltd, Beijing, China; ^6^ Key Laboratory of Vertebrate Evolution and Human Origins, Institute of Vertebrate Paleontology and Paleoanthropology, Center for Excellence in Life and Paleoenvironment, Chinese Academy of Sciences, Beijing, China; ^7^ School of Materials Science and Engineering, Nanjing University of Posts and Telecommunications, Nanjing, China

**Keywords:** goat, genetic diversity, migration, origin, evolution

## Abstract

Despite much attention on the history of goat evolution, information on origin, demographic history, and expansion route remains controversial. To address these questions, we collected 4,189 published goat DNA sequences including 1,228 sequences from 57 breeds in China and 2,961 sequences including 193 goat breeds from 71 other countries and carried out an integrated analysis. We found goat breeds from South China had the highest genetic diversity of lineage B, and subclades B2 only were found in Southwest China, suggesting that lineage B (particularly, subclade B2) probably originated from Southwest China and its surrounding areas. In addition, in this study, we found that lineage A from South China also presented higher genetic diversity and earlier expansion time (10, 606 years ago), even earlier than breeds from the Middle East. Hence, we speculated that South China and surrounding areas were the origin of lineage B and also the transportation hub for lineage A spreading to North China and Southwest Asia. Furthermore, according to the analysis of correlation between genetic differentiation value λ1 and λ2 and geographical distance, we further confirmed two phases of migration in goat breeds of North China. These results will contribute to a better understanding of the origin and migration history of domestic goat.

## Introduction

The domestic goat (*Capra hircus*) was an important globally distributed farm animal that provided indispensable animal products such as meat, milk, hides, and fiber for humans and also played important roles in agriculture and culture for human civilizations (Parma, et al., 2003). Archaeological evidence showed that the Fertile Crescent region of the Near East was the domestication center of domestic goats at about 10,000 years ago (Zeder and Hesse 2000). Meanwhile, recent studies suggest Pakistan was the second, independent domestication center for Cashmere-like goat breeds. Mitochondrial DNA (mtDNA) surveys revealed that goats present different maternal lineages and had undergone population expansion at different times (Naderi, et al., 2007). Recent investigation using mtDNA data discovered six mtDNA lineages in domestic goats. Lineage A was predominant and widely distributed all over the world, whereas lineage B was primarily found in South and Southeast Asia, with their frequency tending to increase southeastward (Lin, et al., 2013; Naderi, et al., 2008). In addition, lineage B was divided into two subclades (B1 and B2), in which subclade B1 is found in a wider geographic area of China and South and Southeast Asia (Li, et al., 2020; Zhong, et al., 2013). Subclade B2 was found only in China and Mongolia (Ganbold, et al., 2020). Lineage C was found in Asia and Europe with low frequencies, and the minor lineage D, F, and G also had limited distribution areas at low frequencies (Naderi, et al., 2007; Tabata, et al., 2019).

Though these previous mtDNA studies had provided useful evidence for the history of goat domestication, answers to important questions on the domestication process were far from being settled. For example, origins of different mtDNA lineages and gene flow between continent-wide patterns remain unaddressed because previous studies only included regional- and subcontinental-scale analysis. For this reason, we collected and downloaded 4,189 mtDNA sequences containing 57 breeds from China, 13 countries in the Middle East, 13 countries in Southwest Asia, 19 countries in Africa, and 26 countries in Europe and carried out an integrated analysis to better understand the genetic diversity, population expansion, and origin of goat mtDNA lineages.

## Methods

A total of 4,189 sequences (covering China, the Middle East, Asia, Africa, and Europe) were obtained from GenBank, including 1,228 sequences from 57 breeds of China and 2,961 sequences including 193 breeds from 71 other countries (Table S1 and Table S5). In addition, six wild goat, *C. cylindricornis* (AJ317870), *C. ibex nubiana* (AJ317871), *C. sibirica* (AJ317874), *C. caucasica* (AJ317875), *C. falconeri* (AB044305), and *C. aegagrus blythi* (AB110590), sequences (Luikart, et al., 2001; Sultana, et al., 2003) and 22 reference individuals of the domestic goat were extracted from GenBank and used for further analysis (Naderi, et al., 2007). All sequences were aligned using the BioEdit software according to a common region of 481 bp that corresponds to the positions 15,707 to 16,187 on the complete goat mtDNA sequence (accession no. AF533441). Haplotype diversity (Hd) and nucleotide diversity (Nd) with their standard errors were estimated by using the program DnaSP v.5.1 (Rozas, et al., 2017). Neighbor-joining (NJ) tree was constructed in Mega 7.1 (Kumar, et al., 2016), with a Kimura 2-parameter model and a bootstrap test (1,000 replications). Mismatch distribution and pairwise-population fixation index (*F*_*ST*_) values were calculated using Arlequin v.3.5 (Excoffier and Lischer 2010). The obtained genetic distances were utilized to draw MDS plots in the R package v. 3.1.2, and eigenvalues (λ) for the first two dimensions (dimensions 1 and 2) were calculated in each breed/population (Lv, et al., 2015). To visualize the geographic distribution patterns of Nd and λ, interpolation maps were constructed using the ArcMap program in ArcGIS v.10.0 software (Darsow, et al., 2009). The inverse distance weighted option with a power of 2 was selected for the interpolation of the surface (Hanotte, et al., 2002; Lv, et al., 2015). We carried out the regression of λ1 and λ2 versus geographic distance from the domestication center of goat (represented by the geographic distance from Kilis Province of Turkey, where ancient domestic goat is located).

## Results

### Geographic Patterns of Goat mtDNA Sequences

**FIGURE 1:**
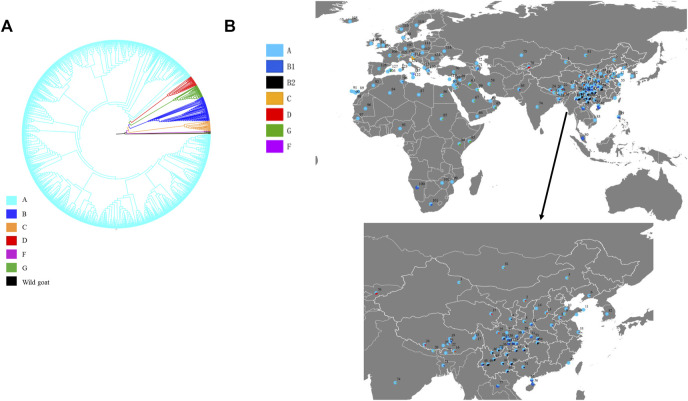
Neighbor-joining trees of domestic goat based on 1,427 mtDNA haplotypes and on the 22 reference mtDNA haplotypes **(A)** and the geographic distribution of domestic goat mtDNA lineages **(B)**. Details of digit code are present in Supplementary Table S1.

**FIGURE 2:**
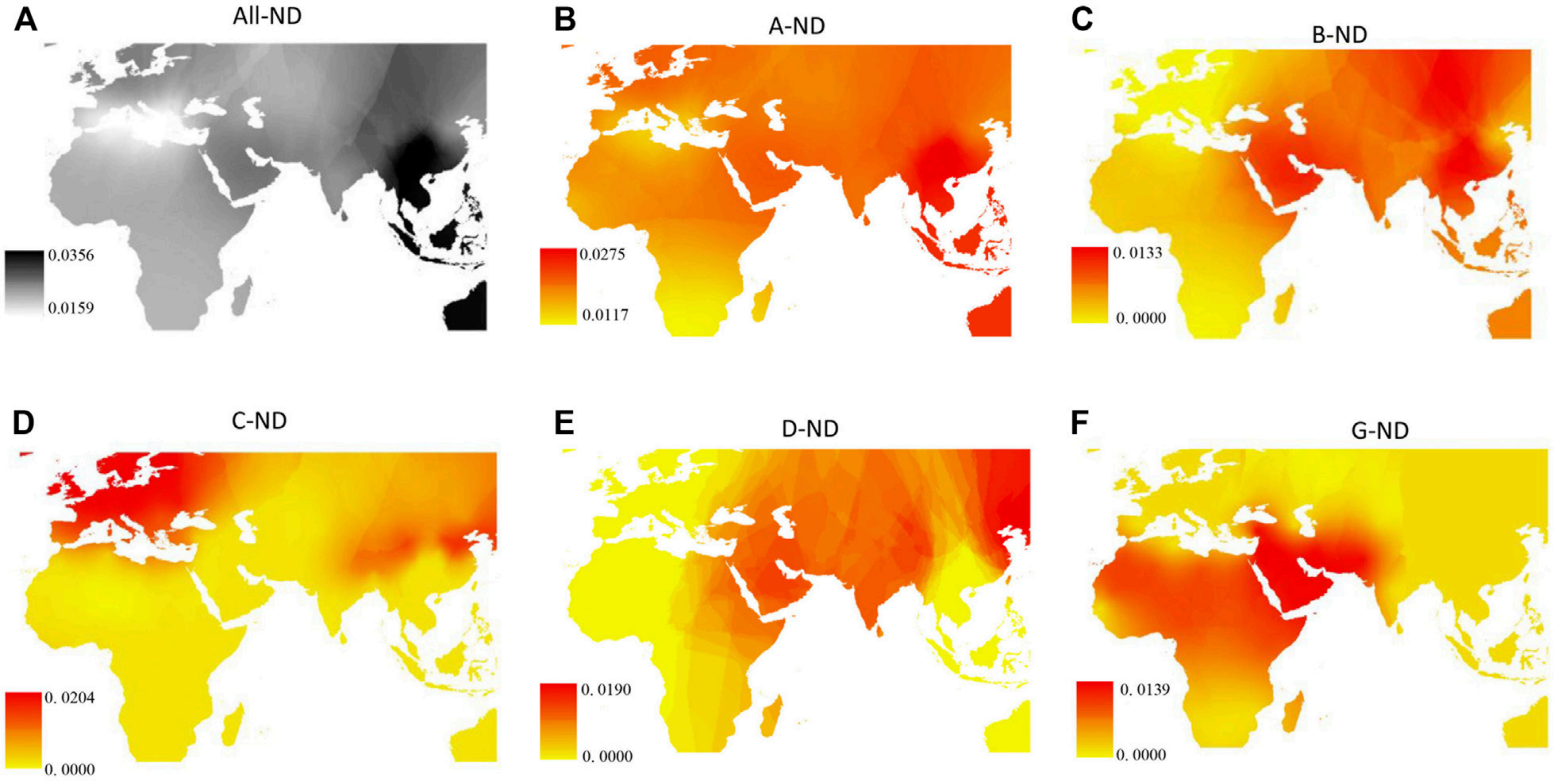
Synthetic maps illustrating the geographic variation of nucleotide variability for the total lineages and goat mtDNA lineages. **(A)** Total lineages; **(B)** **–(F)** lineage A, lineage B, lineage C, lineage D, and lineage G, respectively.

A total of 217 polymorphic sites defining 1,427 haplotypes were detected among 4,189 sequences, and the total haplotype diversity (Hd) and nucleotide diversity (Nd) were 0.9904 and 0.0210, respectively. Phylogenetic analysis constructed based on 1,427 haplotypes and six wild goat sequences in this study presented the six previously defined lineages (A, B, C, D, F, and G) (Figure 1A). Among the six lineages, lineage A was the most common and most widely distributed, with a mean frequency of approximately 89.7% (Figure 1B and Supplementary Table S1). Lineage B was composed of two sub-lineages B1 and B2 previously defined by Chen, et al. (2005). B1 was found mostly in whole Asia, with a few individuals from sub-Saharan Africa and the Middle East and one European goat from Greece, whereas B2 individuals were restricted to China and Mongolia. However, B1 and B2 occurred mainly in South China, especially the Yunnan–Guizhou Plateau and its surrounding areas (Figure 1B and Supplementary Table S1). Lineage C was found in 94 goat individuals of 17 breeds from Asia and Europe at low frequency. Interestingly, lineage C was not found in Africa, and South China only found one individual from YRD breeds. Further analysis of the distribution pattern in China showed that Northern China and Qinghai–Tibet had higher lineage C frequency than South China. Lineage D had the similar distribution patterns with lineage C, also mainly distributed in Northern China and Qinghai–Tibet in the territory of China. Three individuals belonging to lineage F were found only in Sicily. Lineage G was present in the Middle East and Africa, near the Fertile Crescent. In addition, a synthetic map across Asia, Africa, and Europe showed that the breeds in South China had the highest nucleotide variability (Nd), including the all-Nd value, lineage A-Nd value, and lineage B-Nd value (Figures 2A–C). The higher Nd value of lineage C, D, and G was mainly distributed in Europe, Northern China, and Africa, respectively (Figures 2D–F).

### Demographic History of Mitochondrial Lineages

**FIGURE 3:**
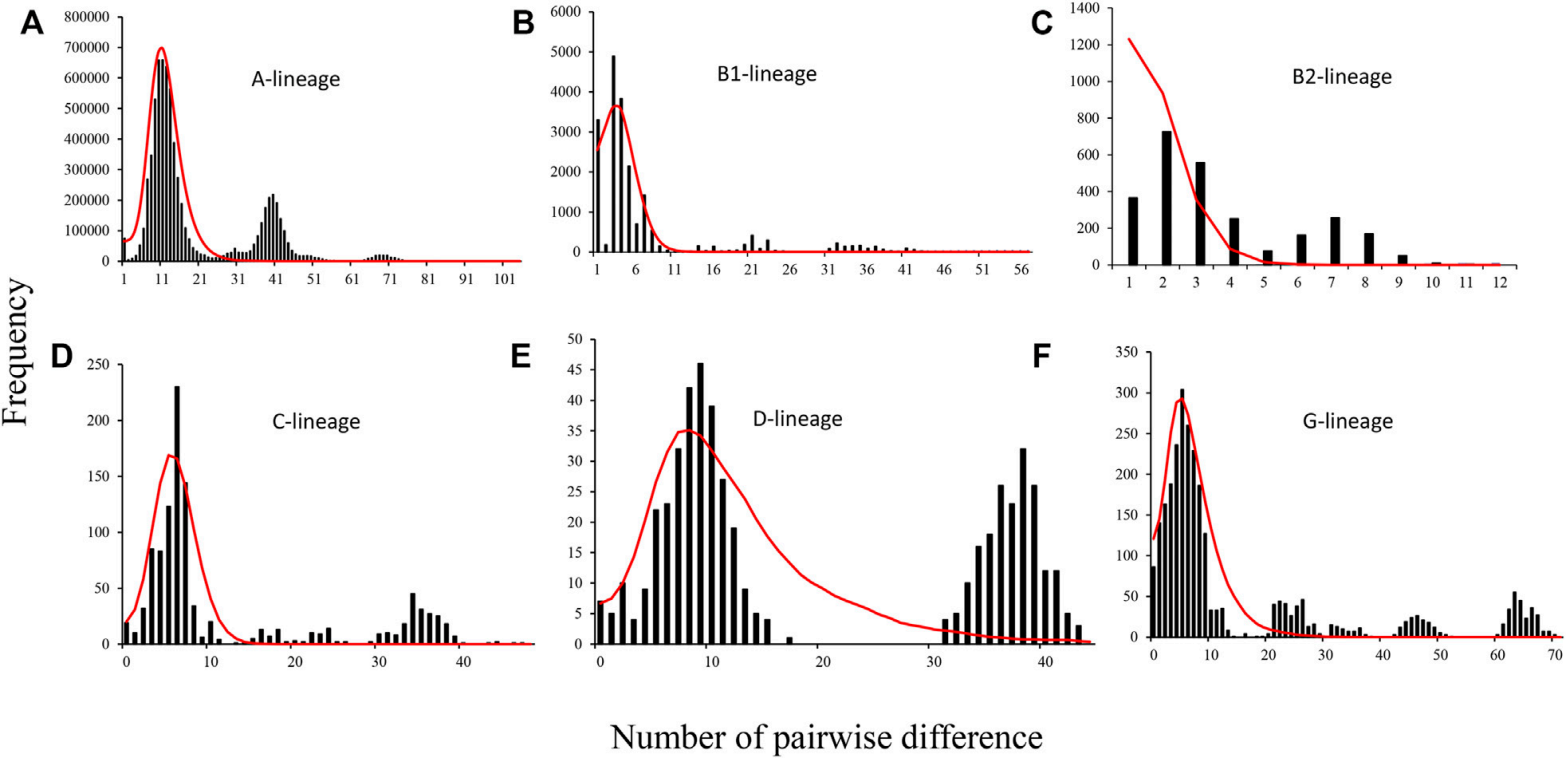
Mismatch distributions for goat mtDNA lineages. **(A)**–**(F)** Lineage A, lineage B1, lineage B2, lineage C, lineage D, and lineage G, respectively. The histogram represents observed values, and the red line represents simulated values.

We estimated the demographic parameters of goat mitochondrial lineages except for the F lineage due to its low frequency. Mismatch distribution results showed a multi-modal distribution for the six mitochondrial lineages (A, B1, B2, C, D, and G) according to the observed values (Figure 3). However, the simulated values from mismatch distribution analysis revealed a unimodal bell-shaped distribution of pairwise sequence differences for all lineages except for B2. In addition, both Fu’s Fs and Tajima’s D statistics showed significant (*p* < 0.05; Supplementary Table S3) departures from neutrality in all lineages. Additionally, the observed mismatch distributions of the lineages were fitted to the sudden population expansion models with very low values of the sum of squared deviation statistic (0.007–0.149)and Harpending’s ruggedness index (0.003–0.046) and all the *p* values >0.05 (Supplementary Table S3). Furthermore, estimation of the postdomestic expansion time for six mitochondrial lineages showed that lineage A had experienced relatively earlier expansion (7.554 ka, 95%CI = 6.246–12.400 ka), and B2 presented the latest expansion (0.683ka, 95%CI = 0.446–5.32 ka 2) (Supplementary Table S3). Lineage C and D had similar expansion time at about 5.418 ka (95%CI = 3.285–6.523 ka) and 5.295 ka (95%CI = 2.587–1.656 ka), respectively. The expansion time for lineage B1 and G was found to be at 2.953 ka (95%CI = 1.162–4.299 ka) and 3.613 ka (95%CI = 1,551–1.269 ka). In addition, expansion time for lineage A in seven major geographic areas (Northern China, Qinghai–Tibet, South China, the Middle East, Southwest Asia, Africa, and Europe) shows that Southern China had much relatively earlier expansions for lineage A (10.606 ka, 95%CI = 7.689–14.650 ka) (Supplementary Table S4). Southwest Asia and Africa had much later expansions of lineage A at 4.328 ka (95%CI = 2.505–15.255 ka) and 4.979 ka (95%CI = 2.858–13.307 ka), respectively. The remaining four areas were found to be approximate the expansion time (Northern China: 9.221 ka, 95%CI = 7.029–10.574 ka; Qinghai–Tibet: 8.469 ka, 95%CI = 7.139–8.962 ka; Europe: 9.351 ka, 95%CI = 6.825–10.921 ka; and the Middle East: 8.615 ka, 95%CI = 7.909–8.951 ka).

### Expansion Route of Postdomestic Goats

**FIGURE 4:**
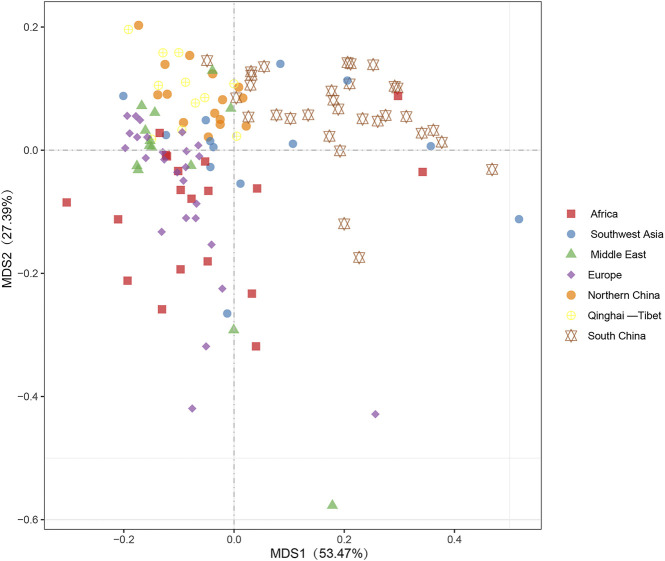
Multidimensional scaling (MDS) plot based on pairwise *F*_*ST*_ values for goat populations from seven geographic areas.

**FIGURE 5:**
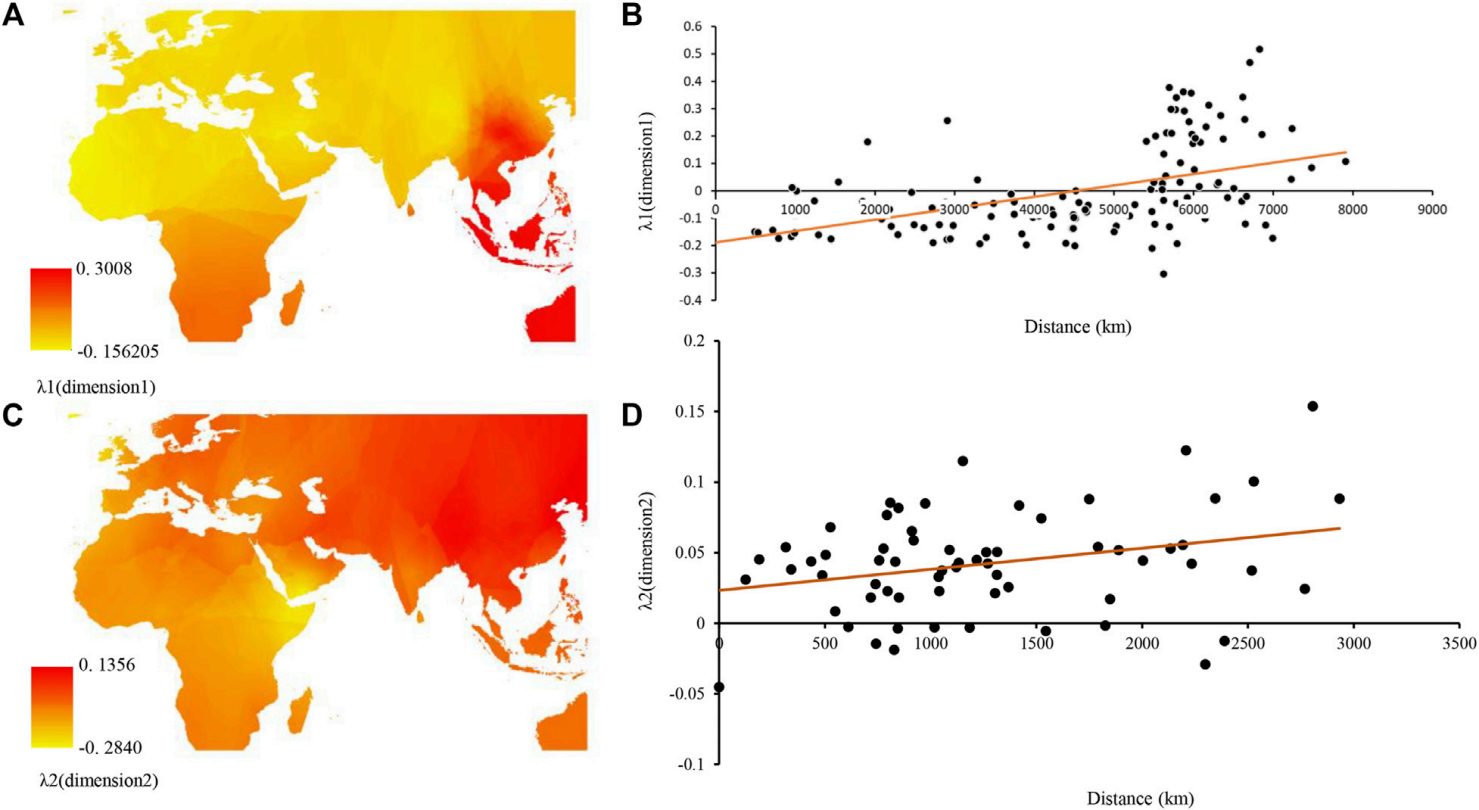
Synthetic maps illustrating the geographic variation of eigenvalues (λ) for the first two MDS dimensions (λ1 and λ2) and regression of λ versus geographic distance from the putative original site of the colonization process. **(A)** Synthetic map for λ1, **(B)** synthetic map for λ2, **(C)** regression of λ1 versus geographic distances from the domestication center of goat (*r* ^*2*^ = 0.22, *p* < 0.0001), and **(D)** regression of λ2 versus geographic distances from a putative “transportation hub” of the Yunnan–Guizhou Plateau (*r* ^*2*^ = 0.08, *p* = 0.02).

Multidimensional scaling (MDS) plot dimension revealed that breeds from Southern China were separated from other breeds and clustered with several individuals from Southwest Asia and Africa, whereas breeds from Northern China and Qinghai–Tibet regions clustered with the Middle East, Southwest Asia, and Europe goat breeds (Figure 4). In addition, both MDS and AMOVA revealed no clear differentiation among geographic regions for goat breeds (Figure 4 and Supplementary Table S2). Furthermore, a synthetic map constructed with the use of interpolated λ1 values and the eigenvalues for the first multidimensional scaling (MDS) plot dimension both showed breeds from Southern China and Africa had higher characteristics of genetic differentiation (Figure A). Additionally, we observed a significant correlation between the λ1 eigenvalues of goat populations and their geographic distances from the domestication center (Figure 5B). This suggested that the major expansion process of domesticated goats in the Middle Eastern to East Asian regions. The interpolation map of the λ2 eigenvalues suggested that the second MDS dimension could represent genetic influence from the Yunnan–Guizhou Plateau region in China. In addition, λ2 explained 27.39% of the total variation and presented a strong and significant correlation observed between geographic distances from the putative region of initial colonization (i.e., the Yunnan–Guizhou Plateau region) and λ2 values across China and Southwest Asia populations (Figures 5C,D).

## Discussion

In this study, we analyzed the mt DNA 4189 sequence variation covering Asia, Africa, and Europe to investigate their maternal origins, population structure, and demographic history **(** Supplementary Table S1 **)**. Six mtDNA lineages described previously in domestic goat were detected according to the results of NJ tree (Naderi, et al., 2007) (Figure 1A). Lineage A was strongly predominant (accounted for more than 90%) and distributed in all breeds (Figure 1B), in agreement with previous studies on goat populations (Tabata, et al., 2019), indicating that lineage A was the main lineage of all modern populations, with the highest frequency. The frequency of lineage A decreases gradually from west to east throughout Eurasia, suggesting that lineage A originated in western Eurasia. However, in this study, we found a higher nucleotide diversity of lineage A in the breeds from Southwest China (Figure 2B), meaning that this region may be a secondary center of dispersal for lineage A, similar with previous studies in sheep from secondary domestication in India (Sachin et al., 2013). The other lineages had more restricted area distributions, for example, lineage B composed of two subclades, B1 and B2. B1 was distributed in the whole of Asia, whereas B2 is found only in China and Mongolia as previously reported (Ganbold, et al., 2020; Li, et al., 2020; Wang, et al., 2015) (Figure 1B, and Supplementary Table S1). Furthermore, given the highest frequency and genetic diversity of lineage B in Southwest China (Figure 1B, Figure 2C), the origin of lineage B may be Southwest China or around its areas, consistent with a number of previous studies (Liu, et al., 2006; Zhao, et al., 2014a; Zhao, et al., 2014b; Zhong, et al., 2013). Lineage C and D had the same distribution pattern and were mainly found in the breeds of the North China, Qinghai–Tibet, Europe, and Middle East regions with a low frequency (Cassidy, et al., 2017; Colli, et al., 2015; Deng, et al., 2018; Ganbold, et al., 2020) (Figure 1B). Interestingly lineage C was absent in African and South China breeds. Based on their geographical distribution, we hypothesized that lineage C and D were spread to North China and Qinghai–Tibet along Central Asia, respectively, after domesticated in the Middle East, similar with a previous study for sheep which spread to East Asia through the Eurasian Steppe (Tabata, et al., 2019) and the Silk Road (Lv, et al., 2015). Meanwhile, lineage C and D were diffused into Southern Europe along the Mediterranean and Danubian route according to the distribution of frequency and nucleotide diversity (Figure 1B, Figures 2D,E). Additionally, lineage G was found only in the breeds of the Middle East and Africa, suggesting that it entered East Africa from Egypt’s Sinai Peninsula via the North African Route, spreading from east to west and south along the North African coast of the Mediterranean as in previous studies (Royo, et al., 2009; Ahmed, et al., 2017). Lineage F was distributed in restricted in Sicily, in agreement with a previous study (Naderi, et al., 2007) (Figure 1B). It was supposed that restricted area distribution for mtDNA lineages may be due to the introgression of wild *Capra* species captured recently and hybridized with domestic goats (Zheng, et al., 2020). In addition, Daly et al. conducted genome-wide analysis of 83 ancient goats from the Paleolithic to Medieval period in the Near East, revealing that several ancient ibex were domesticated in the Near East, resulting in genetically and geographically distinct Neolithic goat populations (Daly, et al., 2018). Additionally, we found there was a weak phylogeographic structure among seven regions (7.63%) (Supplementary Table S2), which was coherent with previously results (Diwedi, et al., 2020; Naderi, et al., 2007; Wang, et al., 2015). Moreover, the genome-wide SNP profiling of worldwide goat populations highlighted a remarkable diversity that occurs at the global scale and was locally partitioned and often affected by introgression from cosmopolitan breeds (Colli, et al., 2015; Colli, et al., 2018). This phenomenon has been explained by the extensive gene flow result in human migration, commercial trade, and extensive transport (Cai, et al., 2020). Furthermore, the MDS plot based on F_ST_ distances also confirmed the mixture in goat breeds of seven regions (Figure 4). However, MDS1 values revealed that breeds of South China had obvious genetic differentiation because of an enclosed environment leading by mountain obstacles and remote geographical location. Furthermore, mismatch distribution and neutrality test analysis both indicated that lineage A, B, C, D, and G had undergone population expansion (Figure 3), being similar with previous studies on domestic goats (Naderi, et al., 2007). Moreover, we found lineage A of South China had the earliest expansion time (about at 10,606 years ago) (Supplementary Table S4). Combined with genetic diversity distribution (Figure 2B), we speculated that southwest of China was a secondary domestication center for lineage A according to previous reports on goat breeds of China, which showed that the Qinghai–Tibet Plateau was an important cradle of Chinese indigenous goats and lineage A of China was possibly derived from Tibetan founders and were dispersed to South China while the others remained (Fan, et al., 2007; Liu, et al., 2006). Therefore, we can come to a conclusion that Southwest China acts as an important “transportation hub” for diffusion of Chinese goats, similar with the study on the migration history of Eastern Eurasian sheep (Lv, et al., 2015). However, lineage B, C, and D expanded relatively more later (B1: 2,953 years ago and B2: 0.683 years ago), indicating a complex process of domestication and migration of goat breeds (Supplementary Table S3).

Moreover, according to findings from this and previous studies, goats from north China arrived from two possible domestication centers through ancient human migration or commercial trade routes (Ganbold, et al., 2020; Li, et al., 2020; Zhao, et al., 2014b). It implied that the first domestication center was the Near East region for north China from lineages A, C, and D whose ancient migration routes could be explained by a migration that started in the Near East region and passed through Central Asia based on the analysis of λ1 eigenvalues versus geographic distances(Figures 5A,B). This migration may have involved ancient sheep population, as since ancient times, nomadic people of North China reared sheep and goat together (Lv, et al., 2015). Second, a domestication center was located close to Southeast China (i.e., the Qinghai–Tibet Plateau and its surrounding areas). We hypothesized that a smaller group of ancient goats from lineage B spread to North China from this center according to the results of regression of λ2 eigenvalues versus geographic distances (Ganbold, et al., 2020) (Figures 5C,D). Furthermore, we found subclade B2 expansion at about 0.683 ka ago (about in the Ming Dynasty) might have spread to north China accompanied by the wars and trade activity (for example, Zhenhe’s traveling to the west) in the Ming Dynasty, whereas subclade B1 arrived at North China at about 2.953 ka ago (about in spring and autumn and Warring States), much earlier than B2 accompanied by human activity and war in spring and autumn and Warring States (Supplementary Table S3).

In summary, the current study provided a comprehensive insight into genetic diversity, distribution pattern, and origin of domestic goats based on world-scale mitochondrial DNA data. Our study detected a relatively high mtDNA diversity in goat breeds from Southwest China, which may be the area of origin for lineage B and also probably was an important goat propagation route to Southeast Asia. In addition, we speculated that two independent domestication centers may have contributed to the formation of goat populations in North China. The results of this study significantly improved our understanding of goat domestication, particularly the dispersal to and from the East Asia region. However, the information based on mtDNA reflected only the female-mediated gene flow. In recent years, with the rapid development of next-generation sequencing technology, nuclear genome research is increasing (Dong, et al., 2013). For example, the whole genome of domesticated goats from all over the world was sequenced and revealed domesticated goats can be divided into East Asia, Southwest Asia, South Asia, Africa, and Europe and also suggested domesticated goats were of not only one origin area. Combined with the ancient data, it was speculated that the domesticated goats were mainly female individuals carrying mitochondrial lineage A and spread to other areas (Zheng, et al., 2020). Moreover, ancient goat genome-wide analysis suggested that Chinese goat originated from ancient western Iran. It was most likely that it originated in The Red Copper Period of Iran (about 6,000–7,000 years ago) and spread to the northwest region about 4,000 years ago.

## Data Availability

The original contributions presented in the study are included in the article/Supplementary Materials, further inquiries can be directed to the corresponding author.
